# Vulnerability to depression is associated with a failure to acquire implicit social appraisals

**DOI:** 10.1080/02699931.2016.1160869

**Published:** 2016-04-06

**Authors:** Andrew P. Bayliss, Steven P. Tipper, Judi Wakeley, Phillip J. Cowen, Robert D. Rogers

**Affiliations:** ^a^ School of Psychology, University of East Anglia, Norwich, UK; ^b^ Department of Psychology, University of York, York, UK; ^c^ Department of Psychiatry, University of Oxford, Oxford, UK; ^d^ School of Psychology, Bangor University, Bangor, UK

**Keywords:** Depression, joint attention, trustworthiness, social cognition

## Abstract

Major depressive disorder (MDD) is associated with disrupted relationships with partners, family, and peers. These problems can precipitate the onset of clinical illness, influence severity and the prospects for recovery. Here, we investigated whether individuals who have recovered from depression use interpersonal signals to form favourable appraisals of others as social partners. Twenty recovered-depressed adults (with >1 adult episode of MDD but euthymic and medication-free for six months) and 23 healthy, never-depressed adults completed a task in which the gaze direction of some faces reliably cued the location a target (valid faces), whereas other faces cued the opposite location (invalid faces). No participants reported awareness of this contingency, and both groups were significantly faster to categorise targets following valid compared with invalid gaze cueing faces. Following this task, participants judged the trustworthiness of the faces. Whereas the healthy never-depressed participants judged the valid faces to be significantly more trustworthy than the invalid faces; this implicit social appraisal was absent in the recovered-depressed participants. Individuals who have recovered from MDD are able to respond appropriately to joint attention with other people but appear to not use joint attention to form implicit trust appraisals of others as potential social partners.

Social isolation is a significant risk factor for depression (Paykel, 1994) while dysfunctional relationships with significant others, friends and peers can help to trigger depressive episodes in vulnerable individuals (Sacco, Milana, & Dunn, 1985). Although social withdrawal during the depressive state itself is very likely mediated, in part at least, by anhedonia and disturbed affect (Blanchard, Horan, & Brown, 2001), the high rates of relapse suggest that particular patterns of interaction with social partners help to bring about the social and affective conditions that promote the onset of depression (Joiner & Coyne, 2002; Segrin, 2000). However, we know relatively little about which disturbances to underlying social-cognitive processes enhance the risks of depressive illnesses in vulnerable individuals.

One way to advance these issues is to examine experimentally how encounters with other people influence social appraisals, or judgements about others as potential social partners, in individuals who have recovered from depression compared to healthy controls with no history of depression. Changes in the formation of such appraisals may tell us something about the psychological mechanisms that mediate social isolation in people who are vulnerable to depression, the nature of the deficits in social skills reported in depressed individuals (Segrin, 2000) and, perhaps, help to identify therapeutic targets.

Shifting one’s attention to objects that other individuals are looking at – establishing “joint attention” – is critical in cognitive and social development (Frischen, Bayliss, & Tipper, 2007), and appropriate responses to such gaze cues is a critical enabler of interactions with others throughout the lifespan (Moore & Dunham, 1995). Previous experiments demonstrate that joint attention can shape implicit judgements or appraisals of other people as potential social partners (Bayliss, Griffiths, & Tipper, 2009; Bayliss & Tipper, 2006). Bayliss and colleagues asked healthy adults to categorise objects that appeared in the left-hand or right-hand side of a computer display (Bayliss et al., 2009; Bayliss & Tipper, 2006). However, just before each object appeared, participants were shown a face whose gaze shifted towards the spatial location at which the object subsequently appeared, or towards the opposite location. Orienting attention in the direction of another person’s gaze to form joint attention is automatic (Driver et al., 1999), so participants were faster to make object discriminations following the presentation of faces whose gaze was directed towards the location of the upcoming objects (“valid faces”) compared to following the presentation of faces whose gaze was directed to the opposite location (“invalid faces”). However, participants subsequently judged the valid faces as more trustworthy than the invalid faces. This induction of pro-social appraisals was implicit, since participants were not consciously aware that some faces reliably indicated the location of the to-be-categorised objects while other faces did not (Bayliss et al., 2009; Bayliss & Tipper, 2006). Nevertheless, participants used this information to form favourable impressions of the valid faces as trustworthy social partners.

There is little evidence to suggest that the *detection* of gaze direction and establishing joint attention is impaired in individuals who have recovered from depression (Gotlib, Krasnoperova, Yue, & Joormann, 2004; Schelde, 1998). Therefore, in this study, we tested the hypothesis that individuals at heightened risk for major depressive disorder, by virtue of having suffered at least two episodes of the illness previously, show normal abilities to follow the eye-gaze of others (and to establish joint attention), but nevertheless fail to acquire implicit trust appraisals of those faces whose gaze enhanced their cognitive performance (valid faces) compared to those faces whose gaze did not (invalid faces). The results show that individuals who have recovered from depression fail to use social cues available in the face to form implicit trust appraisals.

## Methods

### Participants

The study was approved by the Oxford National Health Service (NHS) Research Ethics Committee (08/H0604/62). All participants gave written informed consent. The sample size of 20 in each group was determined prior to the commencement of the study based on previous work of a similar nature in our laboratory. We report all exclusions, manipulations and measures.

Twenty recovered-depressed adults (10 females) and 23 healthy control adults (12 females) took part. Participants were screened using the Structured Clinical Interview for DSM-IV (APA, 2000) and modified Hamilton Depression Rating Scale (HAMD-7) (Hamilton, 1960). They also completed the Beck’s Depression Inventory (BDI) (Beck, Ward, Medelson, Mock, & Erbaugh, 1961) and trait versions of the Positive and Negative Affect Scale (Watson, Clark, & Tellegen, 1988). Cognitive ability was assessed using Raven’s Standard Progressive Matrices (Raven, Raven, & Court, 2004).

The inclusion criteria for recovered-depressed participants included: at least two adult previous episodes of major depressive disorder (MDD); well and medication-free for a minimum of 6 months; a current HAMD-7 score of <7. Exclusion criteria included evidence of low mood within the previous six months, evidence of past or present major psychiatric illness (other than MDD in the case of recovered-depressed participants); a HAMD-7 score of ≥7; taking prescribed medications; past or present DSM-IV alcohol or substance dependence and pathological gambling.

### Stimuli

All stimuli exactly matched those used by Bayliss et al. (2009). Twenty faces from the NimStim face database (http://www.macbrain.org/resources.htm) were arranged in pairs matched for gender, ethnicity and approximate age. One of each pair was designated to Face Group A and the other to Face Group B. The face stimuli comprised two pairs each of black males and black females, and three pairs each of white males and white females. Twelve independent raters ensured that pairs of faces were rated for equal attractiveness and trustworthiness, and that, as a whole, both groups of faces (A and B) were approximately equal in attractiveness and trustworthiness (Bayliss et al., 2009).

The faces measured approximately 10.6 × 10.0 cm. Eye regions measured between 4.0 and 4.5 cm from the left corner of the left eye to the right corner of the right eye. The eyes measured approximately 0.5 × 1.0 cm, with pupils/irises of approximately 0.5 × 0.5 cm. Following Bayliss et al. (2009), all faces held a moderate smiling expression and were initially presented looking straight ahead. Manipulations of the faces allowed the eyes to appear to look towards the right or left. See Supplemental Material for further information about the preparation of the face stimuli.

The target stimuli comprised pictures of 36 household objects. Eighteen objects were categorised as belonging in the kitchen and 18 objects were categorised as belonging in the garage. The objects appeared in red, blue, green or yellow, and in two orientations (e.g. handles of objects on the left or right), yielding 288 stimuli. Targets varied between 1.5–5.0 × 3.0–8.0 cm, and were presented centred 10.0 cm to the left or to the right of the centre of the screen.

### Design and procedure

#### Predictive gaze cueing task

**Figure 1.**
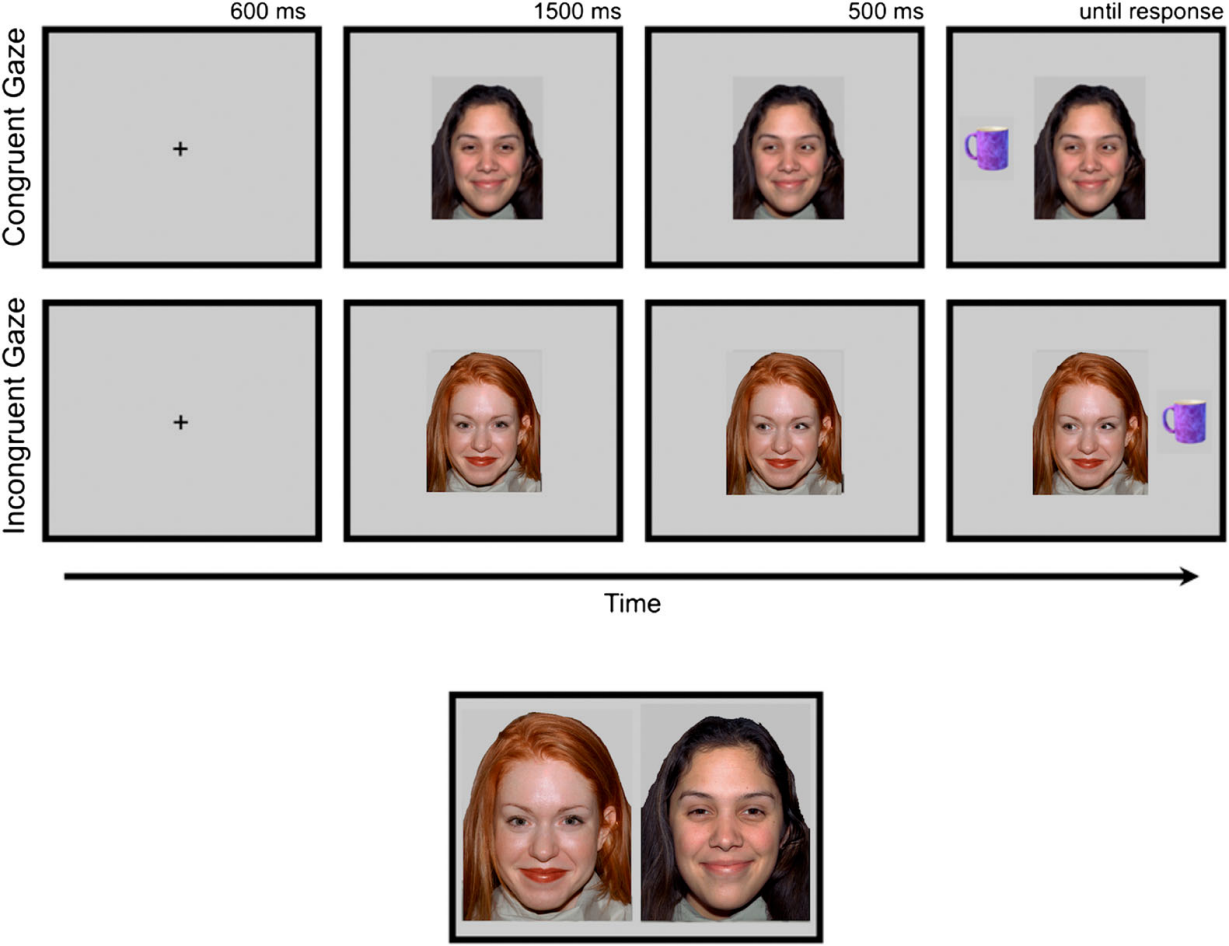
Top panel: trial structure for the gaze cueing task. Valid faces were followed by objects presented on the same side as the shifted gaze; invalid faces were followed by objects on the opposite side. Lower panel: Illustrative example of how face pairs were presented for the trustworthy and memory judgements. Development of the MacBrain Face Stimulus Set was overseen by Nim Tottenham and supported by the John D. and Catherine T. MacArthur Foundation Research Network on Early Experience and Brain Development. Please contact Nim Tottenham at tott0006@tc.umn.edu for more information concerning the stimulus set.

The task has been described previously (Bayliss et al., 2009; Bayliss & Tipper, 2006). Participants were seated, centrally and at an appropriate height, in front of a computer display. Task stimuli were presented at a distance of approximately 60 cm. At the start of each trial, participants fixated a central cross while covering two response keys with the forefinger and thumb of their dominant hand. After 600 ms, the cross was replaced by a face (Figure 1). After another 1500 ms, the eyes of the face moved to the right or left. A household object then appeared 500 ms later, either on the left or right of the display. The participants were instructed to decide, as quickly and accurately as possible, whether the object belonged in the garage (“h” key) or the kitchen (spacebar key). Auditory feedback followed this response (bell = correct, buzzer = incorrect). If no response was made after 2500 ms, the trial was coded as an error, and the next trial was presented. A blank screen was displayed for a 1500 ms inter-trial interval.

Participants completed 12 practice trials using a single novel face. Participants then completed 240 trials (2 blocks of 120), with 10 “valid” (eyes moving towards the target objects) and 10 “invalid” (eyes moving away from the targets) faces appearing 12 times in a random order, paired with randomly selected targets.

Immediately following the gaze cueing procedure, participants completed two binary forced-choice tasks in which they were shown pairs of gender and ethnically matched faces – the same faces that they had viewed in the gaze cueing task. Each pair comprised one “valid” and one “invalid” face, side by side. For each procedure, the order of pairs was randomised, as was the left–right positioning of valid and invalid faces.

#### Trustworthiness

In the first forced-choice procedure, participants were asked to decide which of each pair of faces they felt was more trustworthy by pressing the “1” or “2” keys on the key pad. A blank screen was presented for 2 s after each choice.

#### Memory for faces

In the second forced-choice procedure (“exposure recognition”), participants were asked to decide, in the same way as above, which face of each pair presented they believed had appeared more frequently during the gaze cueing task (due to technical problems, exposure recognition data were not collected for one recovered-depressed participant and three healthy, never-depressed participants).

All participants also then completed another trustworthiness judgements task on a novel, larger set of faces. We did so in order to allow us to explore whether the two groups made similar ratings judgements about faces with whom they had no experience. We briefly present these findings in the “General discussion” section. Finally, all participants were questioned in order to identify and exclude participants who perceived correctly that some (valid) faces in the gaze cueing task shifted their gaze towards the same side of the display as the to-be-categorised object while other (invalid) faces shifted their gaze to the opposite side of the display.

### Statistical analyses

All analyses were carried out using Statistical Package for Social Sciences (SPSS version 14; SPSS Inc., USA). Age, Raven’s Matrices, HAMD-7, BDI, trait positive and trait negative affect scores were each analysed using ANOVA with the between-subject factors of group (recovered-depressed vs. control participants) and gender.

Mean correct reaction times (RTs) and error proportions from the gaze cueing task were analysed using repeated-measures ANOVAs with the between-subject factors of group and gender, and the single within-subject factor of cue (valid vs. invalid faces). Trials with RTs >1500 ms were excluded from the data analysis. Error proportions were arcsine-transformed prior to analysis, though percentage values are reported for convenience.

The proportions of valid over invalid faces chosen in the forced-choice trustworthiness and exposure tasks were analysed using ANOVAs with the between-subject factors of group and gender. Proportions of valid faces chosen by the recovered-depressed and healthy controls were tested using 1-sample *t*-tests against a baseline of chance (5/10 choices of the valid over invalid faces). Finally, associations between choices in the two forced-choice tasks and residual mood symptoms, as measured by the HAMD-7 and the BDI, were tested using Pearson’s correlation coefficients.

## Results

### Group matching and psychometric assessments of mood

Demographic and clinical features of the recovered-depressed and healthy, never-depressed participants are shown in Supplementary Materials (Table S1). The two groups were well-matched in terms of gender, *χ* ^2^(1) < 1, and closely matched for age (*p *= .29) and cognitive ability as measured by Raven’s Matrices (*p* = .19). Trait positive affect was significantly lower in the recovered-depressed participants compared with the healthy control participants, *F*(1, 39) = 6.21, *p *= .017, 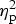 = 0.14; but trait negative affect was significantly higher, *F*(1, 39) = 10.32, *p *= .003, 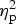 = .22.

As expected, the recovered-depressed participants reported slightly, but significantly, more depressive symptoms as measured by the interviewer-rated HAMD-7 scale, *F*(1, 39) = 15.66, *p *< .0001, 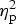 = .29, and BDI, *F*(1, 39) = 13.76, *p *= .001, 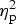 =.26.

### Gaze cueing

Debriefing confirmed that none of the participants in either group were aware of the difference between valid and invalid faces in the gaze cueing task, and that some faces, but not others, consistently cued the location of the to-be-categorised targets.

**Figure 2.**
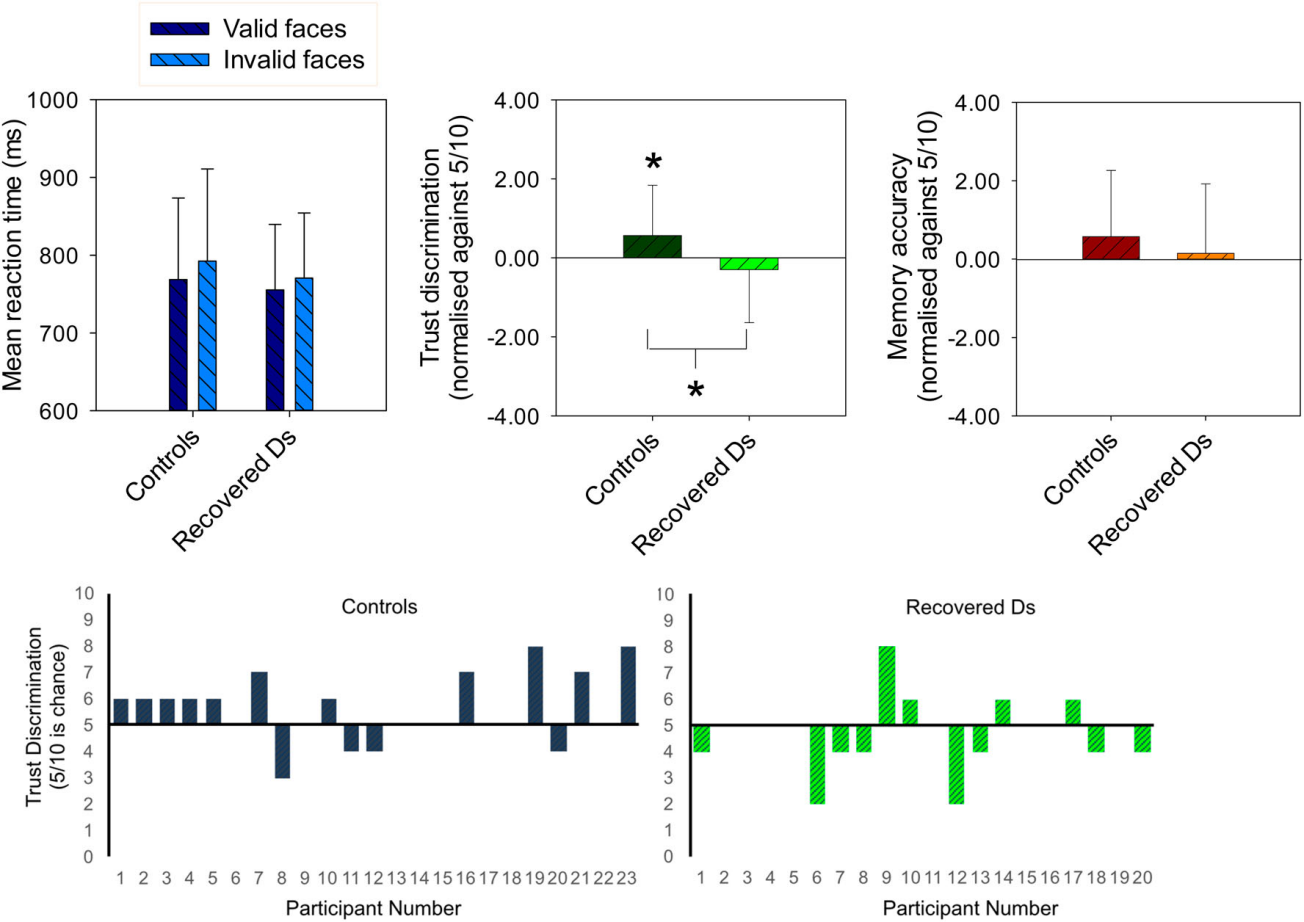
Top panel shows mean correct reaction time (ms ± SD) for target categorisations following valid and invalid faces in the gaze cueing task, and mean number of valid faces chosen as trustworthy and most frequently presented in 20 recovered-depressed adults and 23 age- and ability-matched never-depressed controls. The *y*-axes for the forced-choice trust and memory discriminations are normalised to chance (5/10 correct). *p < .05 from pair-wise and one-sample *t*-tests. Bottom panel shows individual participant data for the critical trustworthiness judgement task (i.e. individual data points that contributed to the central graph of the upper panel of this figure). Chance level performance is where a participant selects five valid cue faces and five invalid cue faces as trustworthy, hence bars above the line indicate that the participant had a bias to select more “valid” gaze cue than “invalid” gaze cue faces.

Error rates were low (<3%; see Supplementary Materials for details). As expected, the mean RTs for categorising kitchen and garage objects were significantly faster following the presentation of valid faces compared with invalid faces (*M*_V_ =762.37 ± 94.99 ms; *M*_In_ =782.21 ± 103.08 ms), *F*(1, 39) = 25.10, *p *< .0001, 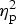 =.39. This was the case for both the recovered-depressed participants, *F*(1, 18) = 8.10, *p *< .05, 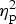 =.31, and healthy never-depressed controls (see Figure 2), *F*(1, 21) = 18.23, *p *< .0001, 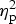 =.47. There was no indication that this facilitatory effect was significantly reduced in the former compared to the latter group of participants, *F*(1, 39) = 1.19, *p *= .29.

Overall, the mean RTs of the recovered-depressed participants were slightly faster than those of the healthy control participants (*M*_RD_ =762.88 ± 83.18 ms; *M*_HC_ =780.48 ± 111.05 ms); however, this difference was not statistically significant, *F*(1, 39) = 0.34, *p *= .56. Male recovered-depressed participants tended to take longer to categorise the targets than female recovered-depressed participants (*M*_MRD_ =811.65 ± 55.56 ms; *M*_FRD_ = 714.10 ± 78.94 ms), while male control participants were slightly faster than female control participants (*M*_MHC_ =766.82 ± 135.11 ms vs. *M*_FHC_ =793.00 ± 87.83 ms). This was reflected in a significant two-way interaction between participant group and gender, *F*(1, 39) = 4.54, *p *= .039, 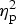 = .10. There were no other significant interactions involving group, gender and cue (all *p*s > .34).

### Trustworthiness discrimination for valid vs*.* invalid faces

When asked to select the more trustworthy faces from pairs of valid and invalid faces, the recovered-depressed participants endorsed significantly fewer valid faces compared to the healthy control participants (see Figure 2), *F*(1, 39) = 4.45, *p *= .041, 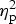 =.10. Replicating previous findings (Bayliss et al., 2009; Bayliss & Tipper, 2006), the healthy controls endorsed significantly more valid faces than chance (5/10; Figure 2), *t*(22) = 2.13, *p *= .045, *d *= 0.45. In contrast, the recovered-depressed participants endorsed slightly fewer valid faces than chance, *t*(19) =−1.0; *p *= .330, *d *= 0.22.

### Memory for valid vs. invalid faces

When asked to select which faces had been presented more frequently, the recovered-depressed participants endorsed a similar number of valid faces compared to the healthy control participants (Figure 2), *F*(1, 37) = 0.04, *p *= .84. Both the healthy controls and the recovered-depressed participants reported seeing the valid faces slightly more frequently than chance (Figure 2); however, neither of these effects were statistically significant, *t*(20) = 1.55, *p *= .137 and *t*(16) = 1.01, *p *= .290, respectively.

### Statistical correlations

Within the recovered-depressed sample, there were no statistically significant associations between residual dysphoric symptoms as measured using the HAMD-7 and BDI scores and choices of valid vs. invalid faces in the forced-choice trustworthy or recognition exposure tasks (−.56 < *r*s(17) < .177).

## General discussion

Social isolation heightens the risk of depressive illnesses in vulnerable individuals (Paykel, 1994) while problematic interactions with social partners can undermine the quality of social contacts that might aid recovery from depression and then protect against relapse (Segrin, 2000). Here, we found that individuals who had recovered from major depressive disorder successfully used interpersonal signals – in this instance, the direction of others’ gaze (as joint attention) – to aid cognitive performance but failed to use these same signals to form implicit trust appraisals. This suggests that people who are vulnerable to depression are not able to extract information from social interactions that would promote judgements of trust in others, perhaps undermining the possibility of durable supporting relationships.

The absence of trust appraisals in our recovered-depressed participants compared with our healthy, never-depressed controls cannot be attributed to differences in gender, age or cognitive ability, as these were carefully matched between the two groups. Our recovered-depressed patients had been well and medication-free for at least six months. Therefore, our results are not explained by the presence of significant depressive illness at the time of testing, or by the psychotropic effects of anti-depressant treatments.

On the other hand, our recovered-depressed participants did report residual dysphoria, and it is possible that these persisting symptoms, expressed in HAMD-7 scores (Hamilton, 1960) and BDI scores (Beck et al., 1961) may be linked to the failure to develop implicit trust appraisals reported here. However, these dysphoric symptoms in the recovered-depressed participants were modest and well-short of published cut-off scores for clinical significance (Hamilton, 1960). Residual dysphoria is also widely observed in samples of individuals with clinical histories of depression (Bhagwagar & Cowen, 2008) and is an important predictor of relapse (Ezquiaga, Garcia, Bravo, & Pallares, 1998). Although the relationship between lowered mood generally and acquisition of trust appraisals remains a target for future investigation, we note that there was no evidence, within our recovered-depressed sample, that low HAM-D or BDI scores were particularly associated with the failures to choose the valid over the invalid faces in the forced-choice trust discrimination task. This suggests that the influence of residual low mood or dysphoric states on the acquisition of trust appraisals was minimal in this data set.

The failure to develop implicit trust appraisals shown by the recovered-depressed participants showed a significant degree of psychological specificity. First, both the recovered-depressed and the matched never-depressed control participants were significantly faster to make object categorisations in spatial locations cued by the valid compared to the invalid faces. In fact, the benefits to performance, in terms of RTs, were almost identical in the two groups while the overall RTs for these decisions were slightly *faster* in the recovered-depressed compared to the healthy control participants. Previous research suggests that individuals with *current* depressive illness can show changes in the maintenance of eye-gaze during dyadic social interactions (Pansa-Henderson & Jones, 1982; Schelde, 1998), perhaps reflecting anhedonia, but that these changes disappear in the euthymic state (Schelde, 1998). Our data suggest that formation of joint attention in response to shifts in other peoples’ gaze is unimpaired in individuals with significant histories of depression but who are not currently unwell.

Second, previous investigations have found that individuals who have recovered from depression show facilitation of memory for negative information following induction of low mood but, in addition, persisting problems with memory for positive information (Gilboa & Gotlib, 1997; Teasdale & Dent, 1987). This raises the possibility that the failure of our recovered-depressed participants to acquire trust appraisals for the valid compared to the invalid faces might reflect poor memory for the former compared to the latter; the valid faces being “tagged” with a positive valence because of their utility in aiding object categorisation through joint attention. However, this is unlikely since, in our experiment, both the recovered-depressed and the healthy never-depressed participants showed a small, but statistically non-significant, tendency to remember the valid faces better than the invalid faces (exposure recognition). Moreover, consistent with evidence that memory deficits for faces are present in dysphoric, but not non-dysphoric, states (Wells, Beevers, Robison, & Ellis, 2010), there was no sign that memory for the valid faces compared to the invalid faces was impaired in the recovered-depressed compared to the never-depressed control participants.

In contrast to their intact ability to follow others’ direction of gaze to form joint attention, our recovered-depressed participants failed to modulate their appraisal of faces whose gaze either enhanced or hindered performance. Replicating previous findings (Bayliss et al., 2009; Bayliss & Tipper, 2006), the never-depressed control participants showed statistically reliable increases in the judged trustworthiness of the valid compared to the invalid faces in the gaze cueing task; however, these trustworthiness judgements were absent in the recovered-depressed participants. Depression often involves the disruption of personal relationships (Segrin, 2000). One explanation for these problems is that they reflect the anhedonia that reduces the reward value of contacts with partners, family and friends (Segrin, 2000). As treatments take effect and symptoms improve, so social contacts are resumed (Blanchard et al., 2001). However, our data suggest that individuals who have suffered depression, but who are now recovered, do not use subtle cues arising from interactions with social partners to form positive or pro-social appraisals – for example those involving trust – that might foster expectations of dependable supportive relationships.

Compared with healthy controls, our participants were less able to learn about the trustworthy behaviour of the “valid” faces. Of relevance, however, is how these individuals explicitly rate faces for trustworthiness in the absence of social cues. Perhaps, for example, faces in general are perceived as untrustworthy, or rate different faces as trustworthy than do healthy controls. To examine this, and to provide additional context to the data we present, we also asked both groups of participants to rate an additional sample of 118 novel faces that they had not viewed in the gaze cueing experiment, and we provide brief details here. Participants rated these faces from −3 (untrustworthy) to +3 (trustworthy).

Overall, there was agreement between the groups, with faces that the healthy controls deemed untrustworthy also receiving low ratings from the recovered depression. Therefore, both controls and recovered-depression participants in this study varied their trustworthiness ratings in broadly similar ways. However, a significant positive linear relationship, *r* = .27, *n* = 118, *p* < .003 was found between the average trustworthiness score for each face with the difference in trustworthiness ratings between the groups (i.e. average score given to a face by the healthy control group minus the average trustworthiness score given by the recovered-depressed group). Hence, as mean facial trustworthiness increases, the recovered-depression group give less favourable appraisals than the healthy controls do. This is an interesting secondary finding as it suggests that as well as failing to use observed behaviour to be able to form trustworthy impressions of individuals after the gaze cueing task, the recovered-depressed group are also somewhat insensitive to the perceptual features in faces that provide cues to high trustworthiness (e.g. Todorov, Baron, & Oosterhof, 2008). It is therefore conceivable that a reticence, by individuals vulnerable to depression, to appraise individuals with high levels of trustworthiness based on no behavioural information (“first impressions”) could be related to a reduced ability to use observed behaviour as a cue to trustworthiness. We therefore simply note this result with interest, as future research will be needed to explore the relationship between trustworthiness judgements on a “first impressions” basis and the ability to learn about behavioural trustworthiness from social cues.

Our findings are relevant to interactionist theories of depression (Joiner & Coyne, 2002). Individuals who are vulnerable to depression sometimes interact with social partners in ways that can increase their isolation (Joiner, Alfano, & Metalsky, 1992). This might involve, for example, repeated attempts to seek reassurance that can disrupt relationships with close partners and family, promoting a sense of disconnection from others and enhancing the risk of illness (Joiner et al., 1992). This behaviour can be triggered by reluctance to trust assurances or loving behaviours that are inconsistent with negative self-schemas (Joiner et al., 1992). Our findings suggest that these problems can arise at an implicit level of processing in which vulnerable individuals fail to use interpersonal signals which, although not consciously noticed in everyday social interactions, can support positive impressions of others that facilitate further contact. The pattern of findings reported here suggest that people with a history of depression produce appropriate behavioural responses to these cues but do not connect them to positive social judgements involving trust.

Further research will be needed to establish whether the problems in using subtle interpersonal signals to develop trust appraisals reflect the cumulative effects of previous depressive episodes or constitute a pre-existing trait. The experience of depression can enhance a variety of risk factors, involving cognitive, social and biological processes that help account for the high rates of relapse (Bhagwagar & Cowen, 2008). Possibly, disrupted interpersonal relationships and social withdrawal consequent to depressive illness induce an enduring failure to form the implicit trust appraisals shown here in the recovered state. On the other hand, prior problems using interpersonal signals to discriminate between social partners who might offer positive relationships, and those who might not, could mean that vulnerable individuals will experience the challenging and less supportive social environments that have been linked to heightened risk of depression and other psychiatric illnesses (Paykel, 1994).

## Conclusions

In summary, the findings reported here indicate that people who are vulnerable to depression by virtue of significant clinical histories of the illness are able to respond appropriately to social cues that facilitate cognitive performance but then fail to use these same signals to form implicit trust appraisals of potential social partners. This change in social cognition may contribute to the deficits in social skills reported in this population and to the social disconnection that promotes recurring illness.

## Supplementary Material

PCEM_A_1160869_Supplementary.docx

## References

[CIT0001] APA (2000). *Diagnostic and statistical manual of mental disorders DSM-IV-TR fourth edition (text revision)*. Washington, DC: American Psychiatric.

[CIT0002] Bayliss A. P., Griffiths D., & Tipper S. P. (2009). Predictive gaze cues affect face evaluations: The effect of facial emotion. *European Journal of Cognitive Psychology*, (7), 1072–1084. doi: 10.1080/09541440802553490 20885988 PMC2945961

[CIT0003] Bayliss A. P., & Tipper S. P. (2006). Predictive gaze cues and personality judgments: Should eye trust you? *Psychological Science*, (6), 514–520. doi: 10.1111/j.1467-9280.2006.01737.x 16771802 PMC2080823

[CIT0004] Beck A. T., Ward C. H., Medelson M., Mock J., & Erbaugh J. (1961). An inventory for measuring depression. *Archives of General Psychiatry*, , 561–571. doi: 10.1001/archpsyc.1961.01710120031004 13688369

[CIT0005] Bhagwagar Z., & Cowen P. J. (2008). ‘It’s not over when it’s over’: Persistent neurobiological abnormalities in recovered depressed patients. *Psychological Medicine*, (3), 307–313. doi: 10.1017/S0033291707001250 18444278

[CIT0006] Blanchard J. J., Horan W. P., & Brown S. A. (2001). Diagnostic differences in social anhedonia: A longitudinal study of schizophrenia and major depressive disorder. *Journal of Abnormal Psychology*, (3), 363–371. doi: 10.1037/0021-843X.110.3.363 11502079

[CIT0007] Driver J., Davis G., Ricciardelli P., Kidd P., Maxwell E., & Baron-Cohen S. (1999). Gaze perception triggers reflexive visuospatial orienting. *Visual Cognition*, , 509–540. doi: 10.1080/135062899394920

[CIT0008] Ezquiaga E., Garcia A., Bravo F., & Pallares T. (1998). Factors associated with outcome in major depression: A 6-month prospective study. *Social Psychiatry & Psychiatric Epidemiology*, (11), 552–557. doi: 10.1007/s001270050093 9803823

[CIT0009] Frischen A., Bayliss A. P., & Tipper S. P. (2007). Gaze cueing of attention: Visual attention, social cognition, and individual differences. *Psychological Bulletin*, (4), 694–724. doi: 10.1037/0033-2909.133.4.694 17592962 PMC1950440

[CIT0010] Gilboa E., & Gotlib I. H. (1997). Cognitive biases and affect persistence in previously dysphoric and never-dysphoric individuals. *Cognition & Emotion*, , 517–538. doi: 10.1080/026999397379881a

[CIT0011] Gotlib I. H., Krasnoperova E., Yue D. N., & Joormann J. (2004). Attentional biases for negative interpersonal stimuli in clinical depression. *Journal of Abnormal Psychology*, (1), 121–135. doi: 10.1037/0021-843X.113.1.121 14992665

[CIT0012] Hamilton M. (1960). A rating scale for depression. *Journal of Neurology, Neurosurgery & Psychiatry*, , 56–62. doi: 10.1136/jnnp.23.1.56 PMC495331 14399272

[CIT0013] Joiner T. E. Jr., Alfano M. S., & Metalsky G. I. (1992). When depression breeds contempt: Reassurance seeking, self-esteem, and rejection of depressed college students by their roommates. *Journal of Abnormal Psychology*, (1), 165–173. doi: 10.1037/0021-843X.101.1.165 1537962

[CIT0014] Joiner T. E. Jr., & Coyne J. T. (2002). *The interactional nature of depression*. Washington, DC: American Psychological Association.

[CIT0015] Moore C., & Dunham P. J. (1995). *Joint attention: Its origins and role in development* . Hove: Psychology Press.

[CIT0016] Pansa-Henderson M., & Jones I. H. (1982). Gaze and gaze avoidance as perceived by psychiatrists during clinical interviews with schizophrenic, depressed, and anxious patients. *Journal of Nonverbal Behavior*, (2), 69–78. doi: 10.1007/BF00986869

[CIT0017] Paykel E. S. (1994). Life events, social support and depression. *Acta Psychiatrica Scandinavica*, , 50–58. doi: 10.1111/j.1600-0447.1994.tb05803.x 8053367

[CIT0018] Raven J., Raven J. C., & Court J. H. (1997). *Mill Hill vocabulary scale: 1998 edition*. Oxford: Oxford Psychologists Press.

[CIT0019] Sacco W. P., Milana S., & Dunn V. K. (1985). Effect of depression level and length of acquaintance on reactions of others to a request for help. *Journal of Personality & Social Psychology*, (6), 1728–1737. doi: 10.1037/0022-3514.49.6.1728 4087144

[CIT0020] Schelde J. T. (1998). Major depression: Behavioral markers of depression and recovery. *Journal of Nervous & Mental Disorders*, (3), 133–140. doi: 10.1097/00005053-199803000-00001 9521348

[CIT0021] Segrin C. (2000). Social skills deficits associated with depression. *Clinical Psychology Review*, (3), 379–403. doi: 10.1016/S0272-7358(98)00104-4 10779900

[CIT0022] Teasdale J. D., & Dent J. (1987). Cognitive vulnerability to depression: An investigation of two hypotheses. *British Journal of Clinical Psychology*, (Pt 2), 113–126. doi: 10.1111/j.2044-8260.1987.tb00737.x 3580646

[CIT0023] Todorov A., Baron S. G., & Oosterhof N. N. (2008). Evaluating face trustworthiness: A model based approach. *Social, Cognitive and Affective Neuroscience*, (2), 119–127. doi: 10.1093/scan/nsn009 19015102 PMC2555464

[CIT0024] Watson D., Clark L. A., & Tellegen A. (1988). Development and validation of brief measures of positive and negative affect: The PANAS scales. *Journal of Personality & Social Psychology*, (6), 1063–1070. doi: 10.1037/0022-3514.54.6.1063 3397865

[CIT0025] Wells T. T., Beevers C. G., Robison A. E., & Ellis A. J. (2010). Gaze behavior predicts memory bias for angry facial expressions in stable dysphoria. *Emotion*, (6), 894–902. doi: 10.1037/a0020022 21058844

