# Supplementary material for: Vulnerability to depression is associated with a failure to acquire implicit social appraisals
Source: Cogn Emot. 2016 Apr 6;31(4):825–33. doi: 10.1080/02699931.2016.1160869 (PMC5415677; doi:10.1080/02699931.2016.1160869)
Supplement: PCEM_A_1160869_Supplementary.docx [file pcem_a_1160869_sm9843.docx]

**Supplemental Materials**

**Further Details on Stimuli**

**Stimuli.** All stimuli exactly matched those used by Bayliss et al 2009 (Bayliss, Griffiths, & Tipper, 2009). Twenty faces from the NimStim face database (http://www.macbrain.org/resources.htm) were arranged in pairs matched for gender, ethnicity and approximate age. One of each pair was designated to Face Group A and the other to Face Group B. The face stimuli comprised 2 pairs each of black males and black females, and 3 pairs each of white males and white females. Twelve independent raters ensured that pairs of faces were rated for equal attractiveness and trustworthiness, and that, as a whole, both groups of faces (A and B) were approximately equal in attractiveness and trustworthiness (Bayliss, et al., 2009).

The faces measured approximately 10.6 x 10.0cm. Eye regions measured between 4.0 and 4.5cm from the left corner of the left eye to the right corner of the right eye. The eyes measured approximately 0.5 x 1.0cm, with pupils/irises of approximately 0.5 x 0.5cm. Following (Bayliss, Schuch, & Tipper, 2010), all faces held a moderate smiling expression and were initially presented looking straight ahead. Manipulations of the faces allowed the eyes to appear to look towards the right or left.

The target stimuli comprised pictures of 36 household objects. Eighteen objects were categorised as belonging in the kitchen and 18 objects were categorised as belonging in the garage. The objects appeared in red, blue, green or yellow, and in 2 orientations (e.g. handles of objects on the left or right), yielding 288 stimuli. Targets varied between 1.5-5.0 x 3.0-8.0cm, and were presented centred 10.0cm to the left or to the right of the centre of the screen (Bayliss, Paul, Cannon, & Tipper, 2006).

**Additional Statistical Analysis**

The mean error rate was less than 3%, and analysis of proportion errors did not yield findings of interest beyond confirming similar performance by both groups in each condition, and so are detailed here for completeness. Participants made marginally more errors when categorising targets following valid faces compared to invalid faces (*E_V_* = 3.06 ± 2.29%; *E_In_* = 2.71 ± 2.29%); however, this difference was not statistically significant, *F*(1,39) = 1.19, *p* = .28. There was no evidence that error rates were differentially altered following valid compared to invalid faces in the recovered-depressed participants (*E_RDV_* = 3.29 ± 2.59%; *E_RDIn_* = 2.87 ± 2.33%) relative to the matched healthy control participants (*E_HCV_* = 2.86 ± 2.05%; *E_HCIn_* = 2.57 ± 2.29%), *F*(1,39) = 0.001, *p* = .98.

Overall, the recovered-depressed participants showed similar error rates compared to the healthy control participants (*E_RD_* = 3.08 ± 2.18%; *E_HC_* = 2.72 ± 1.88%), *F*(1, 39) = 0.59, *p* = .45. Male participants' error rates were slightly elevated compared to female participants' error rates in the recovered-depressed group (*E_MRD_* = 3.58 ± 1.51% *E_FRD_* = 2.58 ± 2.69%) but more closely matched in the healthy control group (*E_MHC_* = 2.80 ± 2.17%; *E_FHC_* = 2.64 ± 1.65%, respectively). However, the two-way interaction between group and gender was not statistically significant, *F*(1,39) =1.80, *p* = .188. There were no other significant interactions involving group, gender and cue (all *p* > .38).

**Table S1.** Demographic and Clinical features (mean±standard deviation) of 20 recovered-depressed adults and 23 never depressed, healthy controls.

| *Group* | *N* | *M:F* | *Age* | *Raven's*  *Matrices* | *HAMD-7* | *BDI* | *Trait +ve affect* | *Trait -ve affect* |
| --- | --- | --- | --- | --- | --- | --- | --- | --- |
| Recovered-depressed | 20 | 10:10 | 31.85±7.42 | 51.60±8.02 | 1.60±1.27 | 3.75±3.21 | 33.35±4.30 | 14.75±3.64 |
| Healthy controls | 23 | 11:12 | 29.70±5.69 | 52.43±4.07 | 0.15±0.71 | 0.96±1.61 | 37.39±3.97 | 11.7±2.22 |
